# Supplementary material for: Faecal Glucocorticoid Metabolites and H/L Ratio Are Related Markers of Stress in Semi-Captive Asian Timber Elephants
Source: Animals (Basel). 2020 Jan 6;10(1):94. doi: 10.3390/ani10010094 (PMC7023510; doi:10.3390/ani10010094)
Supplement: Supplementary file 1 [file animals-10-00094-s001.pdf]

animals

Supplementary Materials: Faecal Glucocorticoid Metabolites and H/L Ratio Are Related Markers of Stress in Semi-Captive Asian Timber Elephants

Martin W. Seltmann [1,*,†], Susanna Ukonaho [1,†], Sophie Reichert [1], Diogo Dos Santos [1], U Kyaw Nyein [2], Win Htut [2] and Virpi Lummaa [1]

[1] Department of Biology, University of Turku, FIN-20014 Turku, Finland; susanna.s.ukonaho@utu.fi (S.U.); reichert.sophie@gmail.com (S.R.); diogo.francodossantos@utu.fi (D.D.S.); virpi.lummaa@gmail.com (V.L.)
[2] Myanma Timber Enterprise, *Ministry of Natural Resources and Environmental Conservation*, Gyogone Forest Compound, Bayint Naung Road, Insein Township, Yangon, Myanmar; kyaw.nyein.mte@gmail.com (U.K.N.); winhtut641@gmail.com (W.H.)
* Correspondence: martsel@utu.fi
† These authors contributed equally to this work.

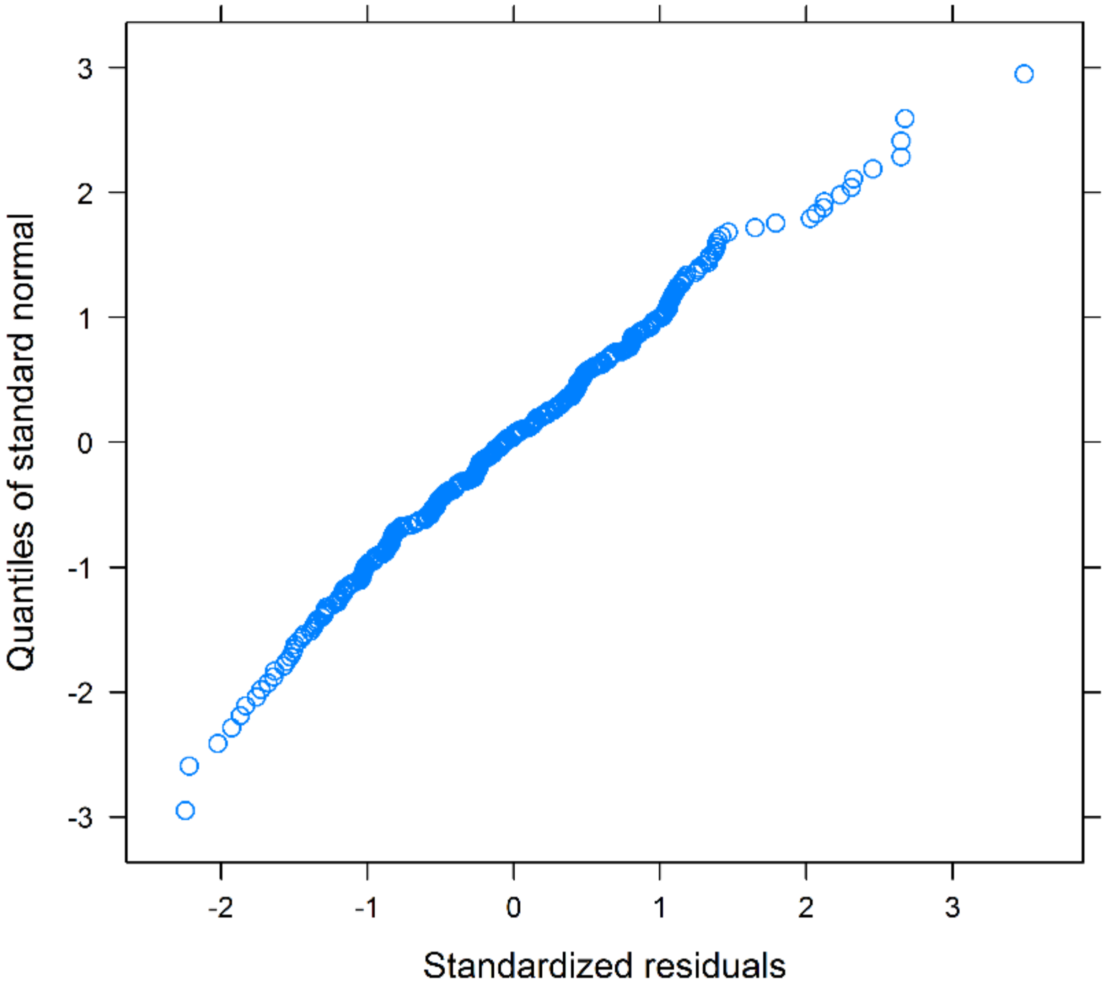

Figure S1. A residual QQ plot indicating model fit for model 1.
